# The Mycobiome: A Neglected Component in the Microbiota-Gut-Brain Axis

**DOI:** 10.3390/microorganisms6010022

**Published:** 2018-03-09

**Authors:** Raphaël Enaud, Louise-Eva Vandenborght, Noémie Coron, Thomas Bazin, Renaud Prevel, Thierry Schaeverbeke, Patrick Berger, Michael Fayon, Thierry Lamireau, Laurence Delhaes

**Affiliations:** 1 Centre de Recherche Cardio-Thoracique de Bordeaux, U1045, FHU ACRONIM, University Bordeaux,33000 Bordeaux, France; louise-eva.vandenborght@genoscreen.fr (L.-E.V.); noemie.coron@chu-bordeaux.fr (N.C.); thomasbazin@club-internet.fr (T.B.); renaud.prevel@hotmail.fr (R.P.); thierry.schaeverbeke@chu-bordeaux.fr (T.S.); patrick.berger@chu-bordeaux.fr (P.B.); michael.fayon@chu-bordeaux.fr (M.F.); thierry.lamireau@chu-bordeaux.fr (T.L.); laurence.delhaes@chu-bordeaux.fr (L.D.); 2 Service d’Exploration Fonctionnelle Respiratoire, Service de Rhumatologie, Service de Gastrologie Adulte, Laboratoire de Parasitologie-Mycologie, CRCM Pédiatrique, Gastroentérologie et Nutrition Pédiatriques, Unité d’Hépatologie, CHU Bordeaux, 33000 Bordeaux, France; 3 INSERM, Centre de Recherche Cardio-Thoracique de Bordeaux, U1045, 33000 Bordeaux, France; 4 Genoscreen Society, 59000 Lille, France

**Keywords:** brain–gut axis, mycobiome, microbiome, dysbiosis, neurological disorders, psychiatric disorders, fungus

## Abstract

In recent years, the gut microbiota has been considered as a full-fledged actor of the gut–brain axis, making it possible to take a new step in understanding the pathophysiology of both neurological and psychiatric diseases. However, most of the studies have been devoted to gut bacterial microbiota, forgetting the non-negligible fungal flora. In this review, we expose how the role of the fungal component in the microbiota-gut-brain axis is legitimate, through its interactions with both the host, especially with the immune system, and the gut bacteria. We also discuss published data that already attest to a role of the mycobiome in the microbiota-gut-brain axis, and the impact of fungi on clinical and therapeutic research.

## 1. Introduction

It has long been accepted that the central nervous system (CNS) and the intestine are closely connected, as suggested by satiety sensations or visceral pains. However, the concept of “microbiome-gut-brain axis (-GBA)” has emerged very recently as a bidirectional communication system in which the digestive microbial flora, also known as the gut microbiome, play a key role [1]. Indeed, accumulated evidence suggests that the intestinal microbiome may modulate CNS activities, which may, in turn, have an impact on the intestinal microbiome [2]. Several studies on mice have illustrated this mutual dialogue well between the gut microbiome and the brain. On the one hand, mice elevated in a sterile environment have an increased anxiety-like behavior that can be reversed after gut colonization with a commensal microbiome [3,4]. On the other hand, the diversity of the gut microbiome is diminished in rodent maternal separation, a model of depression [5]. Lastly, the clinical efficacy of specific probiotic strains in human neuropsychiatric pathologies, such as anxiety or depression, strengthens the concept of the microbiome-GBA [6].

The gut microbiome is a rich and complex ecosystem composed of bacteria, archaea, viruses, fungi, protists, and (sometimes) helminths. The essential role of this ecosystem in host homeostasis, including metabolic and immune functions, is now well demonstrated, as is its involvement in the pathophysiology of digestive and extra-digestive disorders [7,8]. The development of culture-independent techniques for identifying microorganisms, such as next-generation sequencing (NGS), has improved our knowledge on the composition and dynamics of this ecosystem. However, most studies have focused exclusively on the bacterial component, the dominant domain, neglecting fungi and other minority kingdoms [9]. GBA illustrates this trend well since few studies integrate fungal analysis. No review to date has been devoted to the role of intestinal fungi—also named gut mycobiome—in the microbiome-GBA, despite the key role conferred to fungi in digestive diseases [10].

In this review, we summarize the recent findings on the gut mycobiome and its major interactions with the host and the other digestive microorganisms in order to decipher both the existence and the role of a mycobiome-gut-brain axis. Finally, we review the existing literature assessing the links between fungi, the digestive ecosystem, and neurological or neuropsychiatric disorders.

## 2. Gut Mycobiome: State of the Art

Unlike the bacteria that inhabit our digestive tract, the human gut mycobiome has been poorly studied and characterized in healthy as well as in diseased individuals. Initially, the large-scale projects such as the National Institutes of Health’s Human Microbiome Project (HMP) and Metagenomics of the Human Intestinal Tract (MetaHIT) Project were focused exclusively on the bacterial flora to characterize their composition and impact on human health and diseases [11,12]. Bacteria represent huge quantities of microorganisms that inhabit the intestinal mucosa whereas fungi represent a tiny part, estimated at less than 0.01% to 0.1% of genes in stool samples [13,14]. Furthermore, a large part of these fungi are difficult to culture in vitro or are uncultivable [14]. However, the NGS development has been valuable in revealing this poorly understood compartment of our whole microbiome [15]. The main steps of NGS mycobiome analysis are summarized in Table 1 [9,13,15,16,17,18,19,20,21,22,23,24,25].

Since fungi are ubiquitous in our environment—present in the air we breathe, in the food we eat, such as bread, cheese, beer or even in antibiotics—nobody is fungus-free [14,26,27]. Therefore, fungi have been recognized as an integral part of our commensal flora at different body sites (skin, lung, vagina, oral tract, and gut) [28,29]. In the digestive tract, fungi seem to colonize the gut shortly after birth [30,31]. Briefly, the fungal composition of gut flora is influenced by several factors such as age, host genetics, host immunity, diet, and medication [32,33], as well as the bacterial microbiome that also impacts the mycobiome through inter-kingdom interactions [33].

Despite a recent increased number of published data on the gut mycobiome, defining the healthy gut mycobiome is still difficult, especially regarding the high inter- and intra-volunteer variability of the mycobiome. In contrast with gut-associated bacteria, several studies have found a lack of stability in the gut mycobiome over time and low abundance and diversity [13,34]. To date, there is no consensus on the mycobiome “normobiosis,” a term referring to a balanced composition of gut flora in healthy individuals (by contrast, a disruption of this balanced microbial composition of gut flora is named “dysbiosis”). In most studies, Ascomycota is by far the most prevalent fungus phylum in the gut, followed by Zygomycota (corresponding at the previous phylogenetic classification, now distributed among Glomeromycota and several subphyla incertae sedis, including Mucoromycotina, Entomophthoromycotina, Kickxellomycotina, and Zoopagomycotina) and Basidiomycota phyla [33,34,35,36]. Hallen-Adams and colleagues [37] have sequenced stool samples from 45 subjects and observed solely 72 operational taxonomic units (OTUs) assigned as fungal sequences, which is clearly less than bacterial abundance. These OTUs were distributed in two phyla (Ascomycota and Basidiomycota) and in ten classes of micromycetes. The most abundant fungi were *Candida tropicalis* and yeasts belonging to Dipodascaceae. Interestingly, gut fungi observed in this study included known human symbionts (*Candida*, *Cryptococcus*, *Malassezia*, and *Trichosporon* spp.), environmental fungi (*Cladosporium* sp.), and food-associated fungi (*Debaryomyces hansenii*, *Penicillium roqueforti*) [37]. These data reinforce the wide exposure of humans to molds throughout a person’s life. Another NGS study identified 66 fungal genera within 96 stool samples collected from 50 patients, of which 12 were healthy control patients [33]. *Saccharomyces* corresponded to the most prevalent genus followed by *Candida* and *Cladosporium*. A third study observed 75 fungal genera with *Saccharomyces*, *Candida*, and *Penicillium* being the most prevalent [38]. Recently, for the gut mycobiome, Nash and colleagues sequenced 317 stool samples from the American HMP project [13]. Gut-associated fungi in this healthy cohort were mainly composed of a high prevalence of *Saccharomyces*, *Candida*, and *Malassezia*, with *Saccharomyces cerevisiae*, *Malassezia restricta*, and *Candida albicans* being found in 96.8%, 88.3%, and 80.8% of the samples, respectively. Taking together these studies confirms the lower diversity of gut mycobiome in healthy subjects compared to the gut bacterial microbiome [13,33,37,38].

Even if the fungal component is a limited part of the gut ecosystem, it appears to be an essential player of the human microbiome. The increasing interest in gut mycobiome, its dysbiosis, and its role in the GBA is driven by recent data supporting its interactions with the host and the bacterial microbiome.

## 3. Mycobiome Interactions within the Gut Ecosystem

Similar to gut bacteria, the gut mycobiome contributes to physiological functions and homeostasis throughout a host’s lifetime. The effect of the whole microbiome on host health is highlighted by disruptions observed in germ-free mice models [39]. Here we summarize both experimental and clinical data focusing on mycobiome interactions that may be involved in mycobiome-GBA communication through immune and non-immune mediated crosstalk systems, similar to those described in the microbiome-GBA [40].

A first example illustrating the dialogue between fungi and the host immune system is the protective effect of *Saccharomyces boulardii* (the most common probiotic, isolated from fruit) against *Clostridium difficile* colitis. In mice, a prior administration of *S. boulardii* increases the production of immunoglobulin A (IgA), particularly of intestinal anti-toxin IgA [41]. Modulation of the host immune response by *S. boulardii* has also been investigated by Thomas and colleagues [42] who demonstrated that supernatants from *S. boulardii* cultures inhibit the inflammatory response of patients with inflammatory bowel disease (IBD) by inhibiting the activation of T and dendritic cells. The secretion of key pro-inflammatory cytokines such as tumor necrosis factor-α and interleukin(IL)-6 are also reduced [42]. In addition, *S. boulardii* promotes IL-10, an anti-inflammatory cytokine, and epithelial growth factor production [42]. *S. cerevisiae* and *C. albicans* also seem to participate in immune system maturation, inducing functional reprogramming of monocytes and leading to enhanced cytokine production [43,44]. Furthermore, *C. albicans* is able to block monocyte nitric oxide production [44]. This “trained immunity” could be a key factor in the gut immune homeostasis by modulating both the interaction of the host immune system with commensal microorganisms and the host defense against pathogens [45]. Another illustration of this close link between fungi and the immune system is the fungal dysbiosis observed in IBD, an intestinal inflammatory disorder considered as an inappropriate immune reaction against the gut microbiome [46]. Several teams have studied the role of fungal dysbiosis in the pathogenesis of IBD [10,47,48]. To illustrate the fungal impact on IBD, Wheeler and colleagues increased the colitis severity of mice after antifungal administration [48]. Of note, increased plasma levels of (1,3)-β-d-glucan (a major polysaccharide motif of fungal cell walls) are associated with severe colitis in mice [49]. In addition, anti-*S. cerevisiae* antibodies (usually named ASCA) are found to be significantly associated with Crohn disease (CD) in patients [50], which reinforces the concept that fungi are implicated in the inflammatory immune disorder of IBD.

These data highlight the crucial dialogue between the host’s innate immune system and the mycobiome, involving many actors (for review see [51]). Among them, Dectin-1 is one of the most important pattern recognition receptors (PRRs) expressed by immune cells that interact with β-glucan [52]. Dectin-1 knockout mice have more severe colitis compared to wild-type; furthermore, polymorphisms of Dectin-1 gene are associated with increased severity of disease in patients with ulcerative colitis (UC) [53].

Interactions between the gut mycobiome and the host system also influence extra-intestinal immune responses. In mice for example, an antifungal administration induces a disruption of the gut mycobiome, characterized by an expansion of *Aspergillus amstelodami*, *Epicoccum nigrum*, and *Wallemia sebi* and a decrease of *Penicillium brevicompactum* and *C. tropicalis*. In parallel, this fungal dysbiosis is clinically associated with a significant increase in allergic airway disease occurrence, which was confirmed by an increased infiltration of inflammatory cells (mainly eosinophils) into animal lungs [48]. Moreover, fungal supplementation in normobiosis mice with these post-antifungal increased strains (*A. amstelodami*, *E. nigrum*, and *W. sebi*, in order to reproduce the observed post-antifungal dysbiosis) replicated effects similar to allergic airway disease occurrence [48]. Taken together, these results indicate that the commensal mycobiome may be a crucial factor in gut and systemic immunological disorders, based on systemic diffusion of either cytokines, fungal products or metabolites, or micromycetous translocation [49].

On the non-immune mediated crosstalk side and focusing on GBA, fungi are able to synthesize and release neurotransmitters, similar to many bacteria. *S. cerevisiae* and *Penicillium chrysogenum* can produce high concentrations of norepinephrine [54], which is involved in brain activation. This neuromediator increases locomotor activity and aggressive behavior and decreases anxiety reactions. In addition, *C. albicans* is able to produce histamine, another neuromediator involved in appetite regulation, sleep–wake rhythm, and cognitive activity [55]. The direct impact of these mycobiome-produced neuromediators is not entirely clear yet. Even if these neurotransmitters seem unlikely to directly modulate CNS, they could locally act on the enteric nervous system (ENS). Conversely, neuromediators may have an impact on gut fungi. For example, gamma-aminobutyric acid (GABA) is able to increase virulence and germ tube formation of *C. albicans* [56], while serotonin attenuates the *C. albicans* virulence [57].

Finally, inter-kingdom interactions between fungi and bacteria at the gut site may also be implicated in the mycobiome-GBA. While gut bacteria are a known essential actor of the microbiome-GBA [2], mycobiome equilibrium has also been demonstrated as being critical for microbiome stability in a mice model of colitis [49]. In this model, an antifungal exposition induced a fungal diversity decrease along with an increased bacterial diversity, aggravating colitis inflammation and severity [49]. In healthy subjects, Hoffmann and colleagues uncovered specific and significant fungal-bacterial correlations in gut flora [33]. In addition, in the case of bacterial intestinal dysbiosis, such as after antibiotic exposure, commensal fungi or mono-fungal supplementation with *C. albicans* or *S. cerevisiae* can have the same protective benefits as intestinal bacteria in terms of immune system modulation and prevention of mucosal tissue injuries [58]. At molecular levels, *S. boulardii* is able to secrete enzymes, such as proteases or phosphatases, which can inactivate toxins produced by highly inflammatory intestinal pathogens such as *C. difficile* and *E. coli* [59,60]. This yeast also directly inhibits the growth and dissemination of several intestinal pathogens, such as *C. albicans*, *Salmonella typhimurium*, and *Yersinia enterocolitica* [61]. Additionally, β-glucan decreases *E. coli* abundance in animal stools [62], a result suggesting a notable influence of this major fungus wall component on the intestinal growth of *E. coli* and other bacteria, which in turn supports inter-kingdom interactions. On the other hand, *C. albicans* germination is modulated by fatty acids produced locally by bacterial flora [63]. Therefore, we may consider the possibility that the impact of fungi on GBA is due to the local interplay between bacteria and fungi, even though no study has yet focused specifically on this aspect.

These interactions clearly suggest a potential implication of the mycobiome in GBA and, therefore, in various psychiatric and neurological diseases. In the next section, we review evidence of the digestive and neurological aspects of mycobiome influence.

## 4. Mycobiome-Gut-Brain Axis (GBA): Current Evidence from Digestive and Central Nervous Aspects

On the digestive side, many studies have been devoted to the role of gut bacteria in the gut-brain crosstalk. Consistent with this concept, germ-free mice are affected by myelination problems [64] and anxiety-like behavior [65,66], while fecal transplantation modulates this behavior, suggesting once again some strong interactions between the brain and the microbiome [67]. The specific role of gut mycobiome in the communication with the brain is nearly unexplored in the whole microbiome-GBA research field but it is becoming increasingly clear that fungi may have an impact on GBA. Both clinical and experimental data suggest that fungi participate in this dynamic relationship through neuro-immuno-endocrine mediators similar to those described in the microbiome-GBA crosstalk [40]. Numerous evidence consistent with a complex communication network between the gut mycobiome and the brain exists; they are summarized and discussed below.

One main evidence for a key role of the mycobiome in GBA is mycobiome dysbiosis identified in irritable bowel syndrome (IBS). For patients with IBS, a microbiome-GBA disorder is now recognized [68]. It is associated with altered cognitive functions, hypothalamic-pituitary-adrenal axis (HPAA) dysfunctions with lower total cortisol levels, and gut bacterial dysbiosis [69]. In a rat model, antibiotic-induced dysbiosis results in visceral hypersensitivity, a specific clinical trait of rodent IBS models [70]. Furthermore, some studies have shown an efficacy of probiotics in IBS patients [71]. Recently, a mycobiome dysbiosis was also associated with visceral hypersensitivity in a rat model and with human IBS [72], whilst *S. boulardii* supplementation improved gastrointestinal neuromuscular anomalies in a mouse IBS model [73]. These data pave the way for future studies that aim at identifying the specific part of fungi in microbiome-GBA disorders associated with IBS.

Shifts in mycobiome composition have also been reported in various intestinal diseases (for review see [9]), especially in IBD for which an increased fungal load in patients with CD and UC was observed in comparison to healthy controls [10], as well as disease-specific inter-kingdom alterations [10,47,74]. Moreover, depression and psychiatric comorbidities occurred in IBD and have been associated with a systemic inflammation [75]. For example, IL-1 and IL-6 are able to increase cortisol release by HPAA stimulation [76]. Furthermore, patients with depressive disorders exhibit HPAA perturbations with elevated cortisol levels. As previously seen, the mycobiome participates in modulating cytokine production, such as either *C. albicans*, *A. fumigatus* or *S. cerevisiae* with IL-6 [43,77]. Thus, it has been proposed that immune pathways play a critical role here and are mediated by cytokines produced at the gut site, reaching the brain via the bloodstream. These molecules may cross the blood-brain barrier (BBB) and modulate brain area stimulations, particularly the hypothalamus and circumventricular organ stimulations where the BBB is underprovided [78].

In germ-free animals, an increased BBB permeability has been shown, coming from a reduction in tight junction protein expression; this permeability can be decreased after microbial colonization of the mouse digestive tract. BBB permeability is also decreased after gut colonization by short-chain fatty acid (SCFA)-producing bacteria or direct SCFA administration [79]. As fatty acid synthesis fulfills numerous central biological roles in living cells, fatty acid synthase (FAS) is one of the most conserved enzymes of cells including fungal cells. FAS has been described from a variety of yeasts and fungi, such as *S. cerevisiae*, *C. albicans*, other *Candida* species, *Cryptococcus neoformans*, and *Penicillium* species [80]. As fungi such as *S. cerevisiae* or even *Aspergillus fumigatus* are also able to produce short-chain fatty acids [81], we can reasonably hypothesize that the gut mycobiome could use the same pathway; however, no study has explored this hypothesis.

Regarding the central nervous diseases (psychiatric and non-psychiatric ones), the involvement of the human gut mycobiome in the pathophysiology of central nervous diseases has received increased attention in the last few years. The gut mycobiome of an anorexic patient was investigated with culture-dependent and independent approaches. In this case report, the fungal diversity seemed to be decreased with a total of ten different fungal species identified, notably *Aspergillus ruber*, *Penicillium solitum*, *Cladosporium bruhnei* and *Tetratrichomonas* sp. that have not been previously detected in human stools [82].

Another recent study showed for the first time an alteration of the gut mycobiome composition in patients with autism spectrum disorders (ASD), with a trend in increased *Candida* abundance [83]. Given the microbiome influence on ASD based on the increased gastrointestinal problems in this population [84], and given the alteration of BBB permeability and CNS immune response observed in ASD [85] that may be affected by systemic inflammation [86], this over-representation of *Candida* could play a notable role. It may stimulate the host immune response through interactions with specific species such as *Lactobacillus* and may increase IL-22 production [87], a cytokine implicated in the pathogenesis of several autoimmune diseases such as CD and rheumatoid arthritis [88,89]. The *Candida* over-representation may also prevent recovery of a normal flora from a perturbed bacterial flora [90].

Rett syndrome is another progressive neurological genetic disorder often associated with gastrointestinal dysfunctions and constipation. Once again, an increase in the abundance of *Candida* species was described in patients affected by Rett syndrome [91]. In both Rett syndrome and ASD, a predisposition to fungal infections was likewise observed that can, in turn, contribute to systemic responses [92].

Furthermore, a recent study has demonstrated that oral administration of *Candida kefyr* protects mice from developing experimental autoimmune encephalomyelitis, an animal model of multiple sclerosis [93]. This protection was associated with a bacterial microbiome dysbiosis, an increase of regulatory T cells in mesenteric lymph nodes, and a reduction in T-helper 17 cells on the digestive mucosa.

A fungal dysbiosis was found in individuals with schizophrenia, characterized by an increase in *C. albicans* and *S. cerevisiae* species [94,95]. Moreover, the presence of antibody against *C. albicans* is associated with gastro-intestinal disorders as well as lower scores on cognitive tests in these patients [95]. A supplementation with a probiotic formulation composed of *Lactobacillus rhamnosus* and *Bifidobacterium animalis* significantly reduced blood levels of *C. albicans* antibodies and improved psychiatric symptoms [96].

## 5. Concluding Remarks

**Figure 1:**
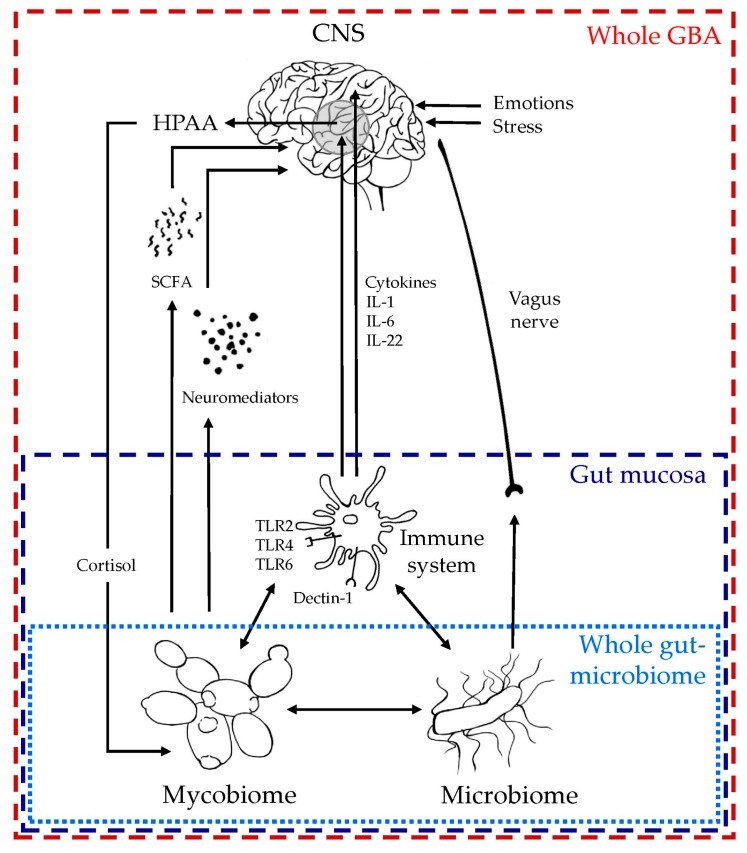
Proposed mechanisms of communication between the gut mycobiome and GBA. Figure inspired from [38,95]; for details about fungi–immune system interactions see review [49,96]. Abbreviations: CNS: central nervous system, GBA: gut–brain axis, HPAA: hypothalamic–pituitary–adrenal axis, IL: interleukin, SCFA: short chain fatty acid, TLR: Toll-like receptor.

It is now well-admitted that the role of the gut microbiome in GBA represents a complex bidirectional system of communication that includes neuro-immuno-endocrine mediators and network pathways between gut mucosa, ENS, and CNS [40,97,98]. In addition to interactions with local bacterial flora and, therefore, acting indirectly on GBA, the gut mycobiome seems to share with the gut microbiome numerous communication processes, which allow us to propose some downward and upward pathways for a mycobiome-GBA in the context of health and disease. These proposed mechanisms of communication between gut mycobiome and GBA are summarized in Figure 1. Furthermore, our growing knowledge on the gut mycobiome, its key role on gut flora equilibrium, and its highly probable role in the whole microbiome-GBA may provide new insight for therapeutic management of neurological and neuropsychiatric disorders such as probiotic administration [73]. In addition, the mycobiome component of gut flora should be systematically taken into account when the gut-microbiome analysis is assessed during clinical trials on GBA.

## Figures and Tables

**Table 1:** Current metagenomic steps to analyze the mycobiome.

Metagenomic Steps	Comments	References
Extraction of fungal communities	- Fungus cell wall is difficult to lyse: mechanical cell disruption (bead beating) or enzymatic cell lysis (lyticase) are usually used; currently, there is no consensus adopted for mycobiome analysis. - Commercial kits are rarely optimized for fungal extraction	[15,16,17]
Libraries preparation	- Metagenomic target debate: Either internal transcribed spacer (ITS1, ITS2) or 18S rDNA are used in mycobiome analysis. In the same study [11], ITS2 and 18S rRNA loci revealed similar results. While 18S primers were able to detect the non-fungal eukaryotic flora, shotgun metagenomics sequencing was in agreement with results from ITS2 sequencing. - Specific NGS method is able to distinguish living and dead cells using pre-treatment with propidium monoazide (PMA)	[8,12,16,18,19,20]
High-throughput sequencing	- Usual sequencing platforms: Illumina (Miseq), Ion Torrent (PGM)) - Uneven ITS length among fungal species may impact species abundance in case of targeted amplicon sequencing - Whole genome sequencing (shotgun metagenomic) may offer both accurate taxonomic assignments and functional data at gene levels but requires higher cost and intensive bioinformatic analysis	[16,18,20,21]
Bioinformatics analysis	- Preprocessing, OTU picking, and taxonomic classification: lack of standardization even if QIIME (Quantitative Insights Into Microbial Ecology), an open-source bioinformatic pipeline, is one of the most used - Quality and completeness of fungal databases lead to different proportions of unassigned sequences (17% of the total OTUs in some studies [11]) - Improving taxonomic assignment quality requires an up-dated fungal database (current databases: Unite, Findley, RTL, TH)	[12,14,16,20,22,23,24]
